# Dural Sac Cross-Sectional Area Measurement as an Indicator of Cauda Equina Syndrome Risk in Patients with Lumbar Disc Herniation

**DOI:** 10.3390/jcm15103906

**Published:** 2026-05-19

**Authors:** Weonmin Cho, Soo-Bin Lee, Young-Seo Park, Kyung-Yil Kang

**Affiliations:** Department of Orthopedic Surgery, Catholic Kwandong University International St. Mary’s Hospital, Incheon 22711, Republic of Korea; science5019@ish.ac.kr (W.C.); 625086@ish.ac.kr (Y.-S.P.); fbdlxk@ish.ac.kr (K.-Y.K.)

**Keywords:** cauda equina syndrome, lumbar disc herniation, dural sac cross-sectional area, risk factor

## Abstract

**Background/Objectives**: Cauda equina syndrome (CES) is a rare but severe complication of lumbar disc herniation (LDH). This study aimed to assess the diagnostic value of the dural sac cross-sectional area (DSCSA) in predicting CES in affected patients. **Methods**: In this retrospective observational study, we analyzed 99 patients who underwent surgery for LDH, including cases with CES, between 2014 and 2023. The DSCSA was measured at the narrowest level of the dural sac using axial T2-weighted magnetic resonance imaging. Univariable and multivariable logistic regression were performed on DSCSA and other candidate risk factors. **Results**: Among the patients with LDH, nine (9.1%) had CES. In multivariable logistic regression, DSCSA was associated with CES, with an adjusted odds ratio of 0.79 (95% confidence interval [CI]: 0.62–0.90, *p* = 0.011). Exploratory receiver operating characteristic analysis identified an optimal DSCSA cut-off of 31.16 mm^2^, yielding 100% sensitivity, 92.2% specificity, and an area under the curve of 0.974 (95% CI: 0.944–1.000, *p* < 0.001). **Conclusions**: Smaller DSCSA was associated with CES in patients with LDH. Patients with a DSCSA of approximately 30 mm^2^ or less may require closer monitoring for the development of CES symptoms. Given the limited number of CES cases, these findings should be interpreted cautiously and validated in larger studies.

## 1. Introduction

Cauda equina syndrome (CES) is a serious and potentially devastating neurological condition that arises when the bundle of lumbosacral nerve roots, collectively referred to as the cauda equina, becomes acutely compressed within the spinal canal. Because these nerve roots innervate the lower extremities, bladder, bowel, and perineal region, CES represents a surgical emergency requiring rapid recognition and intervention. The etiology of CES is diverse, encompassing any pathological process capable of exerting substantial mass effect within the lumbar spinal canal. Major causes include large lumbar disc herniation (LDH), spinal neoplasms, epidural hematomas, infectious processes such as epidural abscesses, and high-energy spinal trauma. Regardless of the underlying cause, the resulting impairment of motor, sensory, and autonomic nerve fibers can lead to profound and irreversible neurological deficits if decompression is delayed [1].

Clinically, CES is characterized by a constellation of symptoms, including severe lower back pain, bilateral or progressive radicular leg pain, urinary retention or overflow incontinence, fecal incontinence or constipation, saddle anesthesia, lower extremity weakness or numbness, and loss of sexual function. Among these manifestations, bladder dysfunction—particularly painless urinary retention—is often regarded as the most specific red flag. However, the clinical presentation may be highly variable, and early or incomplete CES can present subtly, complicating timely diagnosis. Numerous studies have emphasized that the prognosis of CES is strongly time-dependent: immediate surgical decompression offers the best chance of neurological recovery, whereas delays of even several hours may sharply increase the likelihood of permanent deficits, including chronic neuropathic pain, sphincter dysfunction, and paralysis [1,2].

LDH constitutes the most common underlying etiology of CES, accounting for the majority of reported cases. LDH results from displacement of nucleus pulposus material through the annulus fibrosus, typically due to degenerative changes or biomechanical stress [3,4]. Most cases of LDH can be diagnosed using MRI, and functional assessments such as 3D posturography may be useful for evaluating spinal alignment changes associated with LDH as well as alignment alterations before and after surgery [5]. In most patients, LDH produces localized or unilateral compression of individual nerve roots, leading to symptoms such as lower back pain, radicular leg pain, dermatomal sensory loss, and focal motor weakness [6,7]. These typical cases of LDH are generally managed successfully with conservative treatment; however, surgical discectomy may be required in cases that do not respond to non-operative management [5,8].

However, when LDH becomes massive enough to compress not only a single nerve root but the entire dural sac containing the cauda equina, the risk of evolving CES increases substantially. In such scenarios, autonomic dysfunction—including bladder and bowel symptoms, saddle anesthesia, and sexual dysfunction—may rapidly emerge. Importantly, even in the absence of fully developed CES symptoms, severe dural sac compression caused by LDH represents a precarious condition. Emerging evidence suggests that once critical canal compromise is reached, the nerve roots become highly vulnerable to ischemic and mechanical injury, leading to abrupt neurological deterioration [9,10]. For this reason, early surgical decompression should be considered when severe dural sac compression is identified on imaging, even before definitive CES develops, in order to prevent irreversible outcomes. It is well established that delayed diagnosis and treatment can result in irreversible neurological deficits. In the past, specific time thresholds such as 24 or 48 h were proposed, and surgery was recommended within these time frames [1,11]; however, no statistically definitive conclusions could be established. More recently, there has been a shift away from rigid time cut-offs, and instead, immediate surgical decompression as early as possible is generally recommended without reliance on specific time-based thresholds [12].

Despite the clinical significance, relatively few studies have rigorously evaluated predictive markers for CES in the setting of LDH. In particular, quantitative radiologic parameters that could help identify patients at high risk remain insufficiently established. Among these, the dural sac cross-sectional area (DSCSA) measured on axial magnetic resonance imaging (MRI) has attracted attention as an objective indicator of the degree of neural compression. Nonetheless, its diagnostic value for predicting CES risk has not been fully clarified.

In this study, we assessed the utility of DSCSA as a diagnostic and prognostic marker for identifying LDH patients at elevated risk for CES. Additionally, we analyzed supplementary clinical and radiologic variables that may contribute to CES development. Our aim was to establish clinically applicable diagnostic criteria for CES risk in the context of LDH, thereby facilitating earlier recognition, timely surgical decision-making, and potentially better neurological outcomes for patients at risk.

## 2. Materials and Methods

Ethical approval for this study was obtained from the Institutional Review Board of the corresponding author’s hospital (IS23RISI0027). All procedures were conducted in accordance with the Declaration of Helsinki and the institutional guidelines of the corresponding author’s hospital.

### 2.1. Patients

From 2014 to 2023, patients who underwent surgery for central LDH at the authors’ institution were retrospectively analyzed. Patients were divided into two groups: LDH with CES and LDH without CES. Based on widely accepted CES diagnostic criteria [13,14], patients were diagnosed with CES if they had one or more of the following symptoms or signs: (a) bladder and/or bowel dysfunction, (b) reduced sensation in the saddle area, or (c) sexual dysfunction. Patients with foraminal or extraforaminal LDH, which typically does not result in significant dural sac compression, were excluded. To establish an appropriate control group for CES cases characterized by acute massive extrusion, we excluded disc protrusions in which the base occupied ≤ 25% of the disc circumference and the protruded length was shorter than the base [15], as these lesions are predominantly associated with minimal dural sac compression in most cases. In contrast, only disc extrusions, in which the protruded portion extends beyond the base, were included. Patients with CES from other causes, such as tumors, fractures, spinal stenosis, abscesses, or hematomas, were also excluded.

### 2.2. Data Collection

Lumbar spine magnetic resonance imaging (MRI) was performed preoperatively for all patients using a 3T Magnetom Skyra scanner (Siemens, Erlangen, Germany). Axial T2-weighted images were acquired with a 4.00 mm slice thickness, 0.4 mm intersection gap, minimum repetition time of 5000 ms, echo time of 90 ms, 180 mm field of view, echo train length of 15, and a 263  ×  448 matrix.

**Figure 1:**
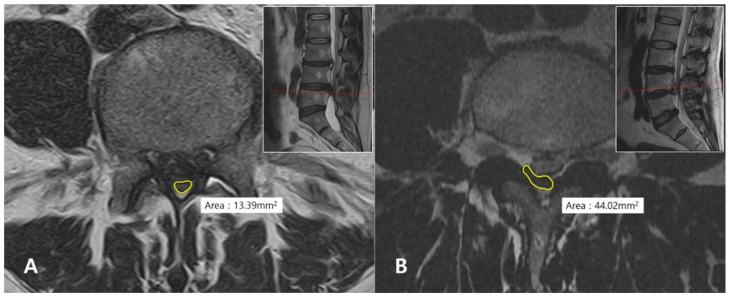
Representative cases showing the measurement of dural sac cross-sectional area (DSCSA). Each figure shows an axial image corresponding to the red line indicated on the sagittal image in the upper right corner. The yellow circle delineates the boundary of the dural sac compressed by the extruded disc. (**A**) Lumbar disc herniation (LDH) with cauda equina syndrome (CES). (**B**) LDH without CES.

The DSCSA was measured at the narrowest point of the dural sac on axial T2-weighted MRI (Figure 1). The measurements were performed using the freehand tool in the INFINITT (Infinitt Healthcare, Seoul, Republic of Korea) picture archiving and communication system software.

The prolapse-to-canal ratio (PCR) was also measured on the same T2-weighted axial images used for the DSCSA assessment. The PCR was calculated as the proportion of the herniated disc area to the inner area of the spinal canal, excluding the ligamentum flavum [16]. All measurements were independently performed by the corresponding author (S.-B.L.), a board-certified orthopedic spine surgeon, and an orthopedic resident (Y.-S.P.). Interobserver reliability for MRI cross-sectional area measurements was assessed using the intraclass correlation coefficient (ICC).

### 2.3. Study Outcome

The primary outcome was to evaluate the association between CES and potential risk factors, including DSCSA. As an exploratory analysis, the secondary outcome was to identify the optimal DSCSA cut-off value and assess its diagnostic performance using receiver operating characteristic (ROC) curve analysis, including the area under the curve (AUC), sensitivity, and specificity.

### 2.4. Statistical Analysis

Differences in baseline characteristics between the LDH with CES and LDH without CES groups were analyzed using the Wilcoxon rank sum test or Fisher’s exact test. Univariable and multivariable logistic regression analysis were conducted on DSCSA and other risk factors. Firth’s penalized multivariable logistic regression was additionally performed to address sparse-event bias. A *p*-value < 0.05 was considered statistically significant. A substantial imbalance in group sizes was observed, reflecting the low incidence of CES in clinical practice and representing a potential statistical limitation. All statistical analyses were performed using R version 4.4.1 (R Foundation for Statistical Computing, Vienna, Austria) in RStudio version 2024.09.0 (RStudio Team, Boston, MA, USA).

## 3. Results

**Figure 2:**
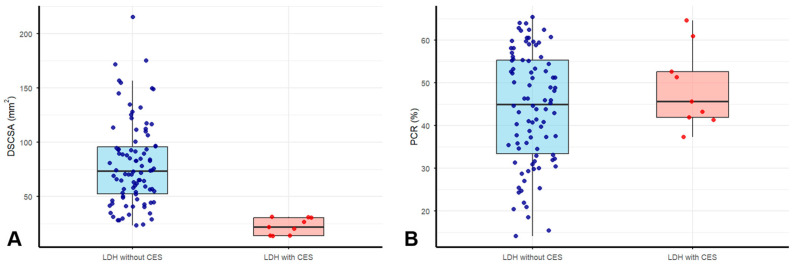
Boxplots with overlaid jittered individual data points showing the distributions of (**A**) DSCSA and (**B**) PCR between the LDH with CES and LDH without CES groups.

Baseline characteristics of the study population are summarized in Table 1. Of the 104 patients who underwent surgery for LDH, 99 met the inclusion criteria and were included in the study. The LDH without CES group comprised 90 patients, while the LDH with CES group included nine patients. All patients with CES were classified as having complete CES (CES-R) with associated urinary retention. The median age was higher in the LDH without CES group (50 years) than in the LDH with CES group (39 years). Height, weight, body mass index (BMI), and the proportion of male patients were higher in the LDH with CES group. The L4–5 level was the most common location for LDH in both groups. The prevalence of L4–5 involvement was higher in the LDH with CES group than in the LDH without CES group (89% vs. 48%). Median DSCSA was significantly smaller in the LDH with CES group compared to the LDH without CES group (21.7 vs. 73.3 mm^2^, *p* < 0.001). PCR was slightly higher in the LDH with CES group; however, there was no statistically significant difference between the two groups. The differences in the distributions of DSCSA and PCR between the two groups are shown in Figure 2. Interobserver reliability was excellent, with ICCs of 0.912 for DSCSA and 0.884 for PCR.

To identify predictive risk factors for CES, univariable logistic regression analyses were performed for each variable, and age, weight, BMI, and DSCSA were found to be statistically significant (Table 2).

Age, BMI, and DSCSA were selected as clinically relevant risk factors for CES in patients with LDH, and multivariable logistic regression analysis was performed. In the multivariable analysis, only DSCSA was significantly associated with CES, with an adjusted odds ratio of 0.79 (95% confidence interval [CI]: 0.62–0.90, *p* = 0.011) (Table 3).

To minimize sparse-event bias related to the limited number of CES cases, we additionally conducted Firth’s penalized multivariable logistic regression analysis. The association between DSCSA and CES remained statistically significant in the penalized model (adjusted OR: 0.85, 95% CI: 0.70–0.93, *p* < 0.001), although the findings should still be interpreted with caution given the small number of CES cases (Table 4).

**Figure 3:**
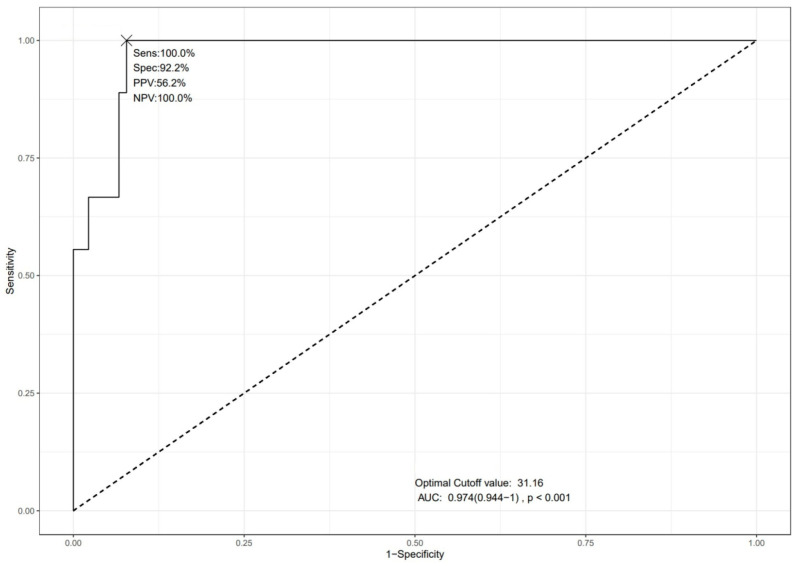
Exploratory receiver operating characteristic (ROC) curve analysis of dural sac cross-sectional area (DSCSA) for cauda equina syndrome (CES) prediction. The diagonal dashed line represents the performance of a random classifier (AUC = 0.5) and is shown as a reference.

Exploratory ROC curve analysis identified an optimal DSCSA cut-off value of 31.16 mm^2^ for predicting CES in patients with LDH, yielding 100% sensitivity, 92.2% specificity, and an AUC of 0.974 (95% CI: 0.944 to 1, *p* < 0.001) (Figure 3).

## 4. Discussion

In this study, we performed a multivariable analysis to explore potential risk factors for CES in patients with LDH, incorporating variables such as DSCSA, age, and BMI, which have been previously suggested in the literature as possible contributors to disease development. Among these factors, DSCSA showed a significant association with CES, suggesting that the degree of dural sac compression may play an important role in the pathogenesis of this condition. To the best of our knowledge, this is the first study to quantitatively evaluate DSCSA specifically in patients with CES and to demonstrate an association between reduced DSCSA and increased CES risk, while also proposing an exploratory diagnostic cut-off value. However, given the limited number of CES cases included in this study, these findings should be interpreted cautiously and require validation in larger cohorts.

Importantly, our results suggested that a DSCSA of less than 31 mm^2^ may be associated with an increased risk of CES in patients with LDH. Although this threshold should be interpreted cautiously given the exploratory nature of the analysis, it may indicate a degree of neural canal compromise at which the cauda equina becomes more susceptible to compression-related injury. From a clinical perspective, DSCSA could serve as a potentially useful quantitative parameter for identifying patients at higher risk of CES before the full clinical manifestation of the syndrome. Nevertheless, further validation in larger prospective cohorts is required before this threshold can be applied in routine clinical decision-making or surgical triage.

CES is a relatively rare but clinically significant condition, with an estimated incidence of 1–3 cases per 100,000 individuals per year [17]. It has been reported to occur in approximately 1–2% of patients with LDH [18,19], highlighting the fact that only a small subset of LDH cases progress to this severe neurological complication. Nevertheless, when CES does occur, the consequences can be devastating. If not promptly recognized and treated with urgent decompression, patients may develop permanent neurological deficits, including lower limb motor weakness, chronic neuropathic pain, sexual dysfunction, and persistent bladder or bowel incontinence. Given the time-sensitive nature of neurological recovery, early prediction and diagnosis of CES in patients with LDH are of paramount importance, emphasizing the need for reliable predictive markers.

Despite its clinical importance, the risk factors for CES remain incompletely understood, largely due to the rarity of the condition and the consequent limitations in study size and statistical power. Previous studies have suggested that CES occurs more frequently in individuals between 30 and 50 years of age [20,21], possibly reflecting the peak incidence of symptomatic disc degeneration in this population. In our study, the median age of the LDH with CES group (39 years) was significantly lower than that of the LDH without CES group (50 years). A possible explanation for this finding may be related to differences in disc hydration and disc height. With increasing age, degenerative changes in the nucleus pulposus lead to reduced hydration and a decrease in intervertebral disc height. Accordingly, in acute disc herniation, a larger volume of herniated material capable of causing severe dural sac compression may be more likely to occur in younger patients in whom the nucleus pulposus is relatively better preserved. In addition, as there was no history of significant trauma in the CES patients in this study; trauma is unlikely to be a major contributing factor to the observed age difference. Further studies are warranted to better elucidate the underlying mechanisms of this finding.

Sex-related differences have also been investigated in the literature; while some reports indicate a higher prevalence in males [22,23], others have failed to demonstrate a significant association, suggesting that sex alone is unlikely to be a decisive factor in CES development [20,21]. Similarly, BMI has been proposed as a potential risk factor due to its association with increased mechanical loading of the spine and accelerated disc degeneration [24,25]. However, conflicting findings have been reported, with some studies showing no significant relationship between BMI and CES risk [16]. In our study, sex was not associated with CES. Although BMI showed a significant association in the univariable analysis, this association was not maintained in the multivariable analysis.

Recently, PCR has been introduced as a radiologic parameter for the early diagnosis and prognostic prediction of CES [16,26,27]. PCR is defined as the ratio of the cross-sectional area of the herniated disc to that of the spinal canal, thereby reflecting the relative degree of canal compromise. Fonseka et al. [27] reported an optimal PCR cut-off value of 66%, with high diagnostic performance (sensitivity 75%, specificity 97%, AUC 0.923), whereas Kaiser et al. [16] suggested a lower cut-off value of 57%, with more modest diagnostic accuracy (sensitivity 74%, specificity 62%, AUC 0.743). These findings indicate that PCR may be useful for risk assessment; however, variability in reported cut-off values and diagnostic performance raises concerns regarding its consistency and generalizability.

In contrast to these PCR-based approaches, our study utilized DSCSA as a direct and absolute measure of dural sac compression. Because CES results from severe compression of the cauda equina within the dural sac, DSCSA provides a more anatomically relevant and intuitive indicator of neural compromise. Unlike PCR, which depends on the relative proportion between disc material and canal size, DSCSA directly quantifies the residual space available for neural elements. This distinction is clinically important because PCR may not accurately reflect the true degree of neural compression when baseline spinal canal size varies among individuals. For example, patients with congenitally narrow canals may develop critical compression at lower PCR values, whereas those with wider canals may tolerate higher PCR values without developing CES.

Historically, a PCR greater than 75% has been considered a key radiologic feature suggestive of CES [28,29]. However, more recent studies have challenged this notion, reporting that only 23–45% of patients with CES exhibit a PCR exceeding this threshold [16,30,31]. This discrepancy underscores the limitations of PCR as a standalone diagnostic metric and highlights the potential influence of inter-individual anatomical variability. In contrast, DSCSA offers a more standardized and reproducible parameter that is less affected by such variability. In our study, the PCR in the LDH with CES group showed a wide distribution, ranging approximately from 37% to 65%, and no statistically significant difference was observed between the LDH with CES and LDH without CES groups. In contrast, DSCSA demonstrated a significant difference between the two groups and remained statistically significant in the multivariable analysis. Furthermore, DSCSA showed favorable exploratory diagnostic performance, with encouraging sensitivity, specificity, and AUC values. These findings suggest that DSCSA may be a more reliable and clinically applicable tool for predicting CES risk.

To our knowledge, this study is the first to propose DSCSA as a practical and rapidly measurable parameter for evaluating CES risk in routine clinical settings. In daily practice, clinicians frequently encounter patients with large disc herniations causing severe dural sac compression on MRI, often accompanied by intense lower back pain and radicular symptoms. However, it remains challenging to determine which of these patients are at imminent risk of progressing to CES. In patients with equivocal clinical symptoms, spine surgeons can easily measure DSCSA using the freehand tool commonly available in PACS software, which may facilitate risk assessment for CES and assist in clinical decision-making regarding treatment strategy.

Nevertheless, this study has several limitations that should be acknowledged. First, due to the low incidence of CES, the sample size was relatively small, which may limit the statistical power and generalizability of our findings. This represents a major limitation of our study, and therefore the statistical findings should be interpreted with caution. Retrospective single-center studies are particularly prone to such limitations, and prospective larger multicenter studies are needed to validate our results across diverse populations. Second, substantial selection bias may be present because the cohort consisted exclusively of surgically treated patients with lumbar disc extrusion, which may have limited the representativeness of the study population and potentially inflated the observed diagnostic performance of DSCSA. Therefore, caution is warranted when generalizing these findings to the broader population of patients with LDH or CES. Third, although PCR was additionally evaluated for comparison, the present data do not allow definitive conclusions regarding the superiority of DSCSA over other radiological parameters, and the comparative findings should therefore be interpreted as preliminary. Fourth, additional radiological parameters, such as fragment migration and congenitally narrow canal, were not analyzed in this study, which may limit the comprehensiveness of the radiological assessment. Finally, only patients with CES-R were included in this study, which may further limit the applicability of the findings to patients with incomplete CES or the broader clinical spectrum of CES.

## 5. Conclusions

Our study demonstrated that DSCSA is significantly associated with the development of CES in patients with LDH. Furthermore, exploratory analyses suggested that DSCSA values around or below 30 mm^2^ may be associated with an increased risk of CES. Given that DSCSA can be measured easily and rapidly in routine clinical practice, it may serve as a practical and clinically useful parameter for assessing CES risk and guiding treatment decisions in patients with LDH presenting with equivocal CES symptoms. Further large-scale prospective multicenter studies are warranted to validate these findings and refine the clinical applicability of DSCSA.

## Figures and Tables

**Table 1:** Baseline characteristics of the study patients with lumbar disc herniation (LDH).

Characteristic	Overall LDH ^1^ (N = 99)	Cauda Equina Syndrome (CES)		*p*-Value ^2^
		LDH Without CES ^1^ (N = 90)	LDH with CES ^1^ (N = 9)	
Age, years	49 (40, 57)	50 (41, 59)	39 (36, 43)	**0.018**
Sex				0.170
Female	46 (46%)	44 (49%)	2 (22%)	
Male	53 (54%)	46 (51%)	7 (78%)	
Height, cm	165 (161, 172)	165 (160, 171)	172 (168, 175)	0.055
Weight, kg	70 (62, 78)	70 (62, 77)	76 (72, 100)	**0.046**
BMI, kg/m^2^	25.7 (22.9, 29.0)	25.7 (22.9, 28.2)	26.6 (25.4, 33.8)	0.146
Smoking				1.000
Never	66 (67%)	59 (66%)	7 (78%)	
Ex-smoker	8 (8%)	8 (9%)	0 (0%)	
Current smoker	25 (25%)	23 (26%)	2 (22%)	
Alcohol				0.714
No	65 (66%)	58 (64%)	7 (78%)	
Yes	34 (34%)	32 (36%)	2 (22%)	
Level of LDH				0.061
L1–2	1 (1.0%)	1 (1.1%)	0 (0%)	
L2–3	2 (2.0%)	2 (2.2%)	0 (0%)	
L3–4	7 (7.1%)	6 (6.7%)	1 (11%)	
L4–5	51 (52%)	43 (48%)	8 (89%)	
L5–S1	38 (38%)	38 (42%)	0 (0%)	
DSCSA, mm^2^	70.0 (43.7, 93.4)	73.3 (52.2, 95.6)	21.7 (14.0, 30.2)	**<0.001**
PCR, %	45.2 (35.0, 55.3)	44.9 (33.5, 55.3)	45.6 (41.9, 52.7)	0.336

^1^ Median (interquartile range); n (%). ^2^ Wilcoxon rank sum test; Fisher’s exact test. *p*-values shown in bold denote statistical significance (*p* < 0.05).

**Table 2:** Univariable logistic regression analysis of predictive risk factors for CES.

Variables	OR	95% CI	*p*-Value
Age	0.93	0.88–1.00	**0.** **037**
Male sex	3.35	0.66–17.00	0.145
Height	1.08	0.99–1.19	0.086
Weight	1.06	1.02–1.10	**0.006**
BMI	1.19	1.03–1.38	**0.019**
Current smoker ^1^	0.73	0.14–3.79	0.711
Alcohol	0.52	0.10–2.64	0.429
L3–4 level LDH ^1^	0.90	0.09–8.48	0.924
DSCSA	0.78	0.65–0.94	**0.0** **09**
PCR	1.01	0.96–1.06	0.742

^1^ Non-smoker and L4–5 level LDH were used as the reference categories, respectively. *p*-values shown in bold denote statistical significance (*p* < 0.05).

**Table 3:** Multivariable logistic regression analysis of predictive risk factors for CES.

Variables	Adjusted OR	95% CI	*p*-Value
Age	0.94	0.81–1.05	0.328
BMI	1.05	0.87–1.32	0.638
DSCSA	0.79	0.62–0.90	**0.0** **11**

*p*-values shown in bold denote statistical significance (*p* < 0.05).

**Table 4:** Firth’s penalized multivariable logistic regression analysis to address sparse-event bias.

Variables	Adjusted OR	95% CI	*p*-Value
Age	0.95	0.84–1.05	0.335
BMI	1.03	0.88–1.25	0.702
DSCSA	0.85	0.70–0.93	**<0.001**

*p*-values shown in bold denote statistical significance (*p* < 0.05).

## Data Availability

The original contributions presented in this study are included in the article. Further inquiries can be directed to the corresponding author.

## Abbreviations

The following abbreviations are used in this manuscript:

CES	Cauda equina syndrome
CES-R	Complete cauda equina syndrome
DSCSA	Dural sac cross-sectional area
CI	Confidence interval
LDH	Lumbar disc herniation
MRI	Magnetic resonance imaging
AUC	Area under the curve
ROC	Receiver operating characteristic
BMI	Body mass index
PCR	Prolapse-to-canal ratio
